# Genome Sequences of Mannheimia haemolytica Serotype A2 Isolates D171 and D35, Recovered from Bovine Pneumonia

**DOI:** 10.1128/genomeA.00093-15

**Published:** 2015-03-12

**Authors:** Melissa J. Hauglund, Fred M. Tatum, Darrell O. Bayles, Samuel K. Maheswaran, Robert E. Briggs

**Affiliations:** a USDA, ARS, National Animal Disease Center, Ames, Iowa, USA; b Department of Veterinary and Biomedical Sciences, University of Minnesota, St. Paul, Minnesota, USA

## Abstract

Here, we report two genomes, one complete and one draft, from isolates of serotype A2 Mannheimia haemolytica recovered from pneumonic bovine lung.

## GENOME ANNOUNCEMENT

Mannheimia haemolytica is a facultative respiratory pathogen of ruminants. Serotype A2 is the most prevalent serotype recovered from the nasopharynx of healthy unstressed calves (1). Under conditions of concurrent virus infection and/or stress, serotypes 1 and 6 selectively proliferate to become more abundant in the nasopharynx and predominate over serotype A2 (2, 3). The predominance of serotypes A1 and A6 in pneumonic disease is likely a reflection of their unique response to host stress rather than disproportionate virulence in lung tissue. Each has been shown to be virulent when delivered directly to the lung (4). Isolates D171 and D35 were recovered from pneumonic calf lung in January of 1984 and December of 1982, respectively. The genome sequencing of these strains was undertaken to further our understanding of the genetic basis of selective bacterial proliferation in the nasopharynx.

The genome sequencing of M. haemolytica strain D171 was achieved using 3 platforms: Roche (454) GS FLX titanium resulting in 27-fold coverage, Illumina GA IIx resulting in 1600-fold coverage, and PacBio RS resulting in 23-fold coverage. Illumina reads were used to error-correct the PacBio reads using CLC-Genomics Workbench v6.0.2. A hybrid assembly using the CLC software was performed and the resultant contigs were aligned to an optical map (OpGen, MapSolver software, Gaithersburg, Maryland) to confirm the assembly and generate a single scaffold. Reiterative alignments of the 454 and corrected PacBio reads >1 kb against the scaffold, using the CLC software, closed all gaps and resulted in a single circular chromosome. The completed D171 genome consists of 2.50 Mb with a G+C content of 41.1%. The draft genome of M. haemolytica D35 was determined using the Roche platform alone which yielded 20-fold coverage. Assembly against the closed D171 reference genome using the CLC software yielded 123 contigs with a total of 2.49 Mb, G+C content of 41.3%, *N*_50_ of 41,579 bp, and 97% of contigs >500 bp.

Annotation of both genomes was accomplished with the NCBI Prokaryotic Genome Annotation Pipeline revision 2.1. Strain D171 contained a total of 2,487 genes including 2,339 predicted protein-encoding genes, 68 frameshifted pseudogenes, 20 rRNA, and 60 tRNA genes. Strain D35 contained a total of 2,513 genes including 2,416 predicted protein-encoding genes, 30 frameshifted pseudogenes, 10 rRNA, and 55 tRNA genes. One clustered regularly interspaced short palindromic repeat (CRISPR) array was detected in each isolate. Coding regions for a number of putative adhesions, transporters, and outer membrane proteins were very divergent or absent in these serotype A2 isolates in comparison to previously reported serotype A1 and A6 isolates. Further study of these divergent gene products may lead to improved understanding of selective nasopharyngeal colonization by M. haemolytica in cattle and new possibilities for disease control.

### Nucleotide sequence accession numbers.

The genome sequence of M. haemolytica strain D171 has been deposited in GenBank under the accession no. CP006573. The genome sequence of M. haemolytica D35 has been deposited in GenBank under the accession no. AUNK00000000. The version described in this paper is version AUNK01000000.
